# Fermentation Characteristics, Antinutritional Factor Level and Flavor Compounds of Soybean Whey Yogurt

**DOI:** 10.3390/foods13020330

**Published:** 2024-01-20

**Authors:** Xinyu Zhang, Jie Long, Jun Liu, Yufei Hua, Caimeng Zhang, Xingfei Li

**Affiliations:** 1 School of Food Science and Technology, Jiangnan University, 1800 Lihu Avenue, Wuxi 214122, China; 6210112123@stu.jiangnan.edu.cn (X.Z.); hunanlongjie@163.com (J.L.); liujun@yuwangcn.com (J.L.); yufeihuajiangnan@126.com (Y.H.);; 2 State Key Laboratory of Food Science and Resources, Jiangnan University, 1800 Lihu Avenue, Wuxi 214122, China; 3 Collaborative Innovation Center of Food Safety and Quality Control in Jiangsu Province, Jiangnan University, 1800 Lihu Avenue, Wuxi 214122, China

**Keywords:** soybean whey, desalination and concentration, flavor compounds, fermentation characteristics

## Abstract

Soybean whey contains high levels of off-flavors and anti-nutritional factors and is generally considered unsuitable for direct application in the food industry. In this work, to reduce beany off-flavors and anti-nutritional factors, and to improve its fermentation characteristics, soybean whey was treated with electrodialysis desalination, vacuum concentration and lactic acid bacteria (LAB) fermentation. The results showed that electrodialysis desalination increased the fermentation rate and the number of viable lactic acid bacteria of soybean whey yogurt. More than 90% of the antinutritional factor level (urease and trypsin inhibitory activity) was removed due to high-temperature denaturation inactivation and LAB degradation. Concentrated desalted soybean whey yogurt (CDSWY) possessed larger values for firmness and consistency, and a denser network microstructure compared with undesalted yogurt. Over 90% of off-flavors including hexanal, 1-octen-3-ol and 1-octen-3-one were removed after electrodialysis desalination and concentration treatment. Meanwhile, the newly generated β-damascenone through carotenoid degradation and 2,3-butanedione improved the pleasant flavor and sensory quality of CDSWY, while the salty taste of CSWY lowered its sensory quality. This study provided a theoretical basis for better utilization of soybean whey to develop a plant-based yogurt like dairy yogurt.

## 1. Introduction

In East Asia, soybeans are used directly as food and are more often processed into tofu, soybean sauce, soybean milk, natto, etc. Soybean whey (SW) is the waste liquid generated during the production of tofu or soybean isolate protein (SPI). For every 1 ton of SPI produced in China, about 20 m^3^ or more of SW is produced [1]. SW has a high biological oxygen demand (BOD), which leads to serious industrial environmental pollution if directly discharged [2]. SW contains a variety of nutrients such as soybean whey protein (SWP), soybean isoflavones, soybean oligosaccharides and saponins. Therefore, the effective utilization of SW can reduce environmental pollution as well as the recovery and utilization of bioactive ingredients.

In recent years, biotransformation methods have been used to treat SW to produce functional beverages. For example, Chua et al. [3] prepared soybean whey beverages using five commercially available non-brewer’s yeast fermenters, which produced different volatile flavors during the fermentation process. Tu et al. [4] prepared a kombucha-fermented SW beverage and found that the antioxidant and antibacterial activity of this beverage had been enhanced. Tu et al. [5] prepared fermented SW beverages using water kefir microbiota, and its nutritional, functional and organoleptic properties were improved. Xu et al. [6] prepared soy protein yogurt using soy protein, SW and soy germ powder as the base material, revealing that the different probiotic compositions affected the quality of the yogurt, the content of estradiol and soy isoflavones. Different from fermented SW beverages, fermented yogurt usually has a high protein content (over 2.0%), while the protein concentration of SW is low (~0.4–0.5%). Thus, SW should be concentrated to meet the required protein concentration, while the antinutritional factors and salt ions are also concentrated during this process, which might have an adverse effect on the quality of fermented soybean whey yogurt. To our knowledge, there are no reports on the fermentation characteristics, anti-nutritional factors and volatile flavor components of yogurt prepared directly from soybean whey.

It was reported that fermented soybean milk had many off-flavors such as a sour and beany smell. The beany odor was mainly derived from hexanal, 1-octen-3-ol, trans-2,4-nonadienal and trans-2,4-decadienal [7,8,9]. As the by-product of SPI, these beany flavors are further enriched in the soybean whey during the SPI process. Microbial fermentation could be used to produce some unique flavors in soybean whey products to mask the soy taste, thus producing a yogurt that was acceptable to consumers [10]. In this study, we assumed that fermentation can reduce beany flavors in fermented soybean whey yogurt.

SWPs mainly consist of six proteins, namely Kunitz trypsin inhibitor (KTI), Bowman–Birk trypsin inhibitor (BBI), lipoxygenase (LOX), beta-amylase, soybean agglutinin (SBA) and cytochrome C [11]. Among them, KTI and BBI are the main anti-nutritional factors. Protein hydrolysis and absorption are inhibited by trypsin inhibitors, and this may lead to pancreatic disease [12]. KTI is heat-sensitive and its activity can be reduced by heat treatment [13]. However, BBI is a heat-stable antinutritional factor, which cannot be easily rendered inactive by heating [14]. As stated above, the protein concentration of SWP (~0.4%) is too low, and it should be concentrated before preparing fermented yogurt. During this process, the salt concentration increases significantly, which is not conducive to the growth and survival of lactic acid bacteria. High salt concentrations can cause an increase in osmotic pressure, thus resulting in damage to the lactic acid bacteria cells [15,16].

In this work, to reduce the level of beany off-flavors and anti-nutritional factors and to improve the fermentation characteristics of soybean whey, desalination and vacuum concentration treatments were used, and the physicochemical properties, antinutritional factor levels and volatile compounds were investigated. Changes in pH, titratable acidity, buffering capacity and viable cell counts of LAB of soybean whey were detected during fermentation. Urease and trypsin inhibitory activity were used to evaluate the anti-nutritional factors of soybean whey and yogurt. Meanwhile, the textural properties, rheological behavior, microstructure, and sensory characteristics of different treated soybean whey yogurts were also studied.

## 2. Materials and Methods

### 2.1. Materials

Defatted soybean meal was purchased from Shandong Yuwang Ecological Food Industry Co., Ltd. (Dezhou, China). Iota-carrageenan was purchased from MSC Co., Ltd. (Yangsan City, Korea). 2-methyl-3-heptanone was purchased from Sigma-Aldrich (Shanghai, China). Commercial lyophilized yogurt starter culture, composed of *Streptococcus thermophilus* (ST) and *Lactobacillus delbrueckii* subsp. bulgaricus (LB), was purchased from Danisco-DuPont (Shanghai, China). The other chemicals were all of analytical grade and purchased from Sinopharm Chemical Reagent Co., Ltd. (Shanghai, China).

### 2.2. Preparation of Soybean Whey, Desalted Soybean Whey and Concentrated Soybean Whey

SW was prepared according to the method by Li et al. [17]. The desalination of SW was carried out using an electrodialysis system (Hebei Sensi Environmental Protection Technology Co., Ltd., Langfang, China) containing a homogeneous anion exchange membrane pack at 24 V for 1 h. The electrical conductivity of SW before and after electrodialytic treatment was ~8.2 mS/cm and ~1.4 mS/cm, respectively. The desalted soybean whey was defined as DSW.

One and a half liters of SW and DSW were put in glass bottles and concentrated using a rotary evaporator (R-501 rotary evaporator, Shanghai Shenshun Biotechnology Company, Shanghai, China) in a water bath at 75 °C under 0.1 MPa for 1.2 h to obtain concentrated SW and DSW, which were defined as CSW and CDSW, respectively.

### 2.3. Preparation of Soybean Whey Yogurt

The prepared CSW and CDSW were used as base material, then 0.5% oil (*w*/*w*), 0.5% carrageenan (*w*/*w*), 0.1% sodium citrate (*w*/*w*) and 0.1% disodium hydrogen phosphate (*w*/*w*) were added, respectively. After sterilization at 95 °C for 10 min, and cooling to 42 °C, 0.008% (*w*/*w*) starter culture containing *Streptococcus thermophilus* (ST) and *Lactobacillus delbrueckii* subsp. bulgaricus (LB) was added to start fermentation. The inoculated concentrated whey was fermented at 42 °C for about 8 h until the pH dropped to about 4.5. All fermentation samples were rapidly cooled to end the fermentation and refrigerated for 12 h at 4 °C before analysis. Two types of yogurts were obtained, concentrated soybean whey yogurt and concentrated desalted soybean whey yogurt, defined as CSWY and CDSWY.

### 2.4. pH, Titratable Acidity (TA), Buffering Capacity and Viable Cell Counts of LAB

The pH value of each yogurt sample was measured in triplicate using a digital pH meter (Mettler Toledo, Griefensee, Switzerland) at 20 °C. The TA was determined by adding 0.1 mol/L sodium hydroxide solution to a 30 g sample containing 10 g of yogurt and 20 g of cooled distilled water. The volume of sodium hydroxide solution consumed for the titration to pH 8.3 was recorded and the TA was calculated according to the following equation:

$$\mathrm{TA}=\frac{c\times V\times 100}{m\times 0.1}$$

where TA is the titratable acidity in units of ◦T, c is the concentration of NaOH solution (mol/L), V is the volume of NaOH consumed (mL) and m is the weight of the sample (g).

The buffering capacity was measured based on the method reported by Dashper et al. [18] with some modifications. CSW was diluted to a protein concentration of 2.5% (*w*/*w*) and samples (40 mL) were titrated with 0.1 mol/L hydrochloric acid to pH 3.9 at 25 °C with continuous stirring. The volume of hydrochloric acid consumed per 0.1 change in pH was recorded. The following equation was used to calculate buffering capacity:

$$\frac{\mathrm{dB}}{\mathrm{dpH}}=\frac{V_{2}\times C_{\mathrm{HCl}}}{V_{1}\times \Delta \mathrm{pH}}$$

where V_1_ is the volume of the sample in mL; V_2_ is the volume of hydrochloric acid standard solution consumed in mL; C_HCl_ is the molar concentration of the hydrochloric acid standard solution in mol/L; and ΔpH is the change in pH of the sample before and after the titration of the hydrochloric acid standard solution.

Viable cell counts were determined by the plate counting method, and MRS medium (Basebio Technology Co., Ltd, Hangzhou, China) was used to prepare the agar media for LAB counting. Yogurt samples were diluted with 0.90% sterile sodium chloride solution, 7 dilutions of each sample were prepared, and each dilution was divided into triplicates and incubated at 36 ± 1 °C for 72 ± 2 h. Medium with colony counts ranging from 30 to 300 was selected for counting and the results were expressed as colony-forming units per gram of cells (CFU/g).

### 2.5. Composition Analysis

#### 2.5.1. Total Solids, Protein and Sugar

The total solids content of the samples was determined using the direct drying method. The protein content was determined with the bicinchoninic acid (BCA) method according to Rogatsky et al. [19]. Oligosaccharides in SW were analyzed as previously reported by Wang et al. [20] with some modifications. The analysis of oligosaccharides was performed with HPLC. Each sample was analyzed in triplicate.

#### 2.5.2. SDS-PAGE

SDS-PAGE was performed to determine the protein composition according to Wu et al.’s method [21]. The samples were diluted with deionized water to a protein concentration of 2 mg/mL. A 16% separation gel and a 4.0% stacking gel were used. A 0.5 mL quantity of sample solution was mixed with an equal volume of sample dissolution buffer (4.0% SDS, 20.0% glycerol, 0.125 mol/L Tris-HCl buffer pH 6.8). Dithiothreitol (DTT) was added (20 μL) as a reducing agent and 0.01% bromophenol blue was added (10 μL) as an indicator. Ten microliters (μL) of each sample were loaded onto an SDS-PAGE gel and electrophoresed at a constant current of 45 mA and a maximum constant voltage of 30 V. The maximum constant voltage was adjusted to 100 V as the first band ran exactly to the demarcation line between the separation gel and the stacking gel. The electrophoresis was terminated when the first band happened to run out of the bottom of the gel. The gels were scanned using a computing densitometer (Molecular Imager Chemi Doc XRS+, Bio-Rad, Hercules, CA, USA).

### 2.6. Texture Properties

The textural property of each yogurt was measured using the back extrusion method described by Kaur et al. [22] with slight modifications. Measurements were carried out in triplicate using a TA.XT. plus texture analyzer (Stable Micro Systems Ltd., Surrey, UK) equipped with a 25 mm cylindrical probe. The results were calculated using TPA-macro in the software program Texture Expert exceed Version 1.0 (Stable Micro Systems software) to obtain hardness, consistency, cohesiveness and index of viscosity.

### 2.7. Microstructures

The microstructure of soybean whey yogurt was observed by confocal scanning laser microscopy (CLSM, Carl-Zeiss-Promenade, Jena, Germany). A 1 mL quantity of yogurt was mixed with 20 μL of rhodamine B (2 mg/mL). After half an hour of staining, approximately 3 mL of the stained sample was dropped onto a concave glass slice and covered with a glass coverslip. The excitation wavelength of the microscope used was 568 nm, the objective parameters were 40×/NA0.85 and the scanning density was 1024 × 1024.

### 2.8. Activity of Urease and Trypsin Inhibitors

#### 2.8.1. Urease Activity

The urease in the sample was qualitatively determined using the Nessler method. It is based on the color development reaction of ammonium with mercury-containing reagents [23]. Briefly, 0.1 g of sample was mixed with 1 mL of urea solution (10 g/L) and placed in a water bath at 40 °C ± 1 °C for 20 min. No urea was added as a blank control. Then, 4 mL of water, 1 mL of sulfuric acid solution (50 mL/L) and 1 mL of sodium tungstate solution (100 g/L) were added and mixed evenly. After filtration, the filtrate was collected and mixed with 15 mL of water and 1 mL of sodium potassium tartrate (20 g/L), and then 2 mL of Nessler reagents were added and shaken well. The color of the solution in the cuvette was observed over a period of 5 min. If the sample tube was a brick-red cloudy or clarified liquid, it was strongly positive for urease. If the sample tube was an orange-red clarified liquid, it was sub-strong positive. If the sample tube was a dark golden yellow or yellow clarified liquid, it was positive. If the sample tube was a pale yellow or slightly yellow clarified liquid, it was weakly positive. If the sample tube was the same color or lighter than the blank control tube, it was negative.

#### 2.8.2. Trypsin Inhibitory Activity (TIA)

Trypsin inhibitory activity was measured as previously reported by Lu et al. [24]. BAPA was dissolved in 0.05 mol/L Tris-HCl buffer (pH 8.2, containing 1% (*v*/*v*) dimethyl sulfoxide and 0.02 mol/L CaCl_2_). A 1 mL quantity of sample and 2.5 mL of BAPA solution were mixed, then 1 mL of 0.01% (*w*/*v*) trypsin solution was added for an exact reaction of 10 min, then 0.5 mL of 30% (*v*/*v*) acetic acid was quickly added to terminate the reaction. Absorbance was measured at 410 nm. The following equation was used to calculate the TIA of the sample:

$$\mathrm{TIA}=\frac{A_{\mathrm{standard}}-A_{\mathrm{inhibition}}}{0.01}$$

where 0.01 is a unit of trypsin inhibitor activity, defined as reducing the absorbance of the reaction system by 0.01, and A_standard_ is the absorbance value of the reaction system without the addition of inhibitor. A_inhibition_ is the absorbance value of the reaction system after the addition of an inhibitor.

### 2.9. Volatile Compounds

Volatile compound analysis was performed according to the method by Achouri et al. [25] with some modifications. The volatiles were analyzed using gas chromatography–mass spectrometry (GC-MS, 1200 L, Varian, Palo Alto, CA, USA) with a DB-WAX column (0.25 μm, 30 m × 0.25 mm, Agilent, Santa Clara, CA, USA). Thirty grams of sample were accurately weighed and 16% (*w*/*w*) NaCl solution was added, stirred well and fixed to 100 mL with water. A 20 mL quantity of internal standard (2-methyl-3-heptanone, 32 μg/mL) and 5 mL of sample were added to a 20 mL glass vials. Volatiles were characterized by comparing the results of the searches with standard compounds from mass spectrometry databases (NIST and WILEY databases). Each sample was taken in triplicate.

### 2.10. Rheological Analysis

Time sweep: Dynamic oscillatory rheological measurements were conducted with a rheometer (MCR301, Anton Paar, Graz, Austria) equipped with parallel plate geometry (PP50, 50 mm diameter and 1 mm gap) following the procedures by Wang et al. [20]. Simulating the whole process of yogurt production: acidification stage (at 42 °C until pH 4.6), cooling stage (from 42 to 4 °C at a 1 °C/min rate) and annealing stage (at 4 °C for 1 h).

Frequency sweep: Frequency sweep (0.01~10 Hz, 0.1% strain) was carried out at 25 °C to characterize the viscoelastic properties of different yogurt samples. G′ and G″ were plotted against frequency in a logarithmic ramp.

### 2.11. Sensory Evaluation

The sensory characteristics of the soybean whey yogurt samples were evaluated by 8 trained panelists. The panel consisted of 4 females and 4 males, aged 23~26 years, with an average age of 25 years. Two types of soybean yogurt samples were provided in identical-looking plastic cups and randomly coded with three digits. The test forms consisted of four sensory attributes: color (15 out of 15), taste (30 out of 30), odor (30 out of 30) and texture (25 out of 25). The total score was 100 out of 100, with higher scores representing a higher level of acceptance of the sample.

### 2.12. Statistical Analysis

General linear models and least significant difference (LSD) tests were performed using SPSS version 27.0 (Cary, NC, USA) for statistical analysis. *p* < 0.05 was considered a significant difference.

## 3. Results and Discussion

### 3.1. Basic Composition of Soybean Whey after Desalination and Concentration

Electrodialysis refers to the selective permeability of ion exchange membranes to ions in solution under the action of an applied direct current field, causing ion migration of anions and cations in solution to pass through anion and cation exchange membranes, respectively, thus achieving the goal of desalination [26]. As shown in Table 1, the solid content of SW was decreased after desalination because the salt was removed by electrodialysis. In addition, the loss of protein and carbohydrate components was also observed during the electrodialysis processing because of the inevitable adsorption loss on the membrane. In terms of sugar composition, the oligosaccharides in SW mainly consisted of sucrose and stachyose, with fructose and raffinose being present in relatively small amounts. The content of all four oligosaccharides was reduced after desalination, but sucrose and stachyose still dominated. Owing to the low protein content in SW and DSW, SW and DSW were concentrated using a vacuum rotary evaporator in order to achieve the required concentration for fermentation (2.5%). After concentration, CDSW contained more oligosaccharides compared with CSW; due to the higher concentration, multiple were required for DSW to reach the same protein content.

The commercial LAB used in this study were dominated by *Lactobacillus bulgaricus* and *Streptococcus thermophilus*, and the corresponding counts of LAB for CSWY and CDSWY at different fermentation times are shown in Table S1 (Supplementary Materials). After fermentation for 8 h, the number of LAB in CSWY was 1.9 × 10^7^ CFU/g, which was lower than that at 0 h (2.2 × 10^7^), suggesting an inhibiting effect on the growth of LAB. In contrast, the number of LAB increased by an order of magnitude for CDSWY (3.7 × 10^8^ CFU/g), indicating the good growth of LAB in CDSWY. It was supposed that the high concentration (2.37%) of salt in CSW could inhibit the growth of LAB by causing damage to the lactic acid bacteria cells since both samples had similar sugar contents but very different salt contents. The above results also show that DSW and CDSW can provide effective carbon sources for the growth of LAB without extra sugar addition.

### 3.2. Fermentation Characteristics of Soybean Whey after Desalination and Concentration

#### 3.2.1. Buffering Capacity

**Figure 1:**
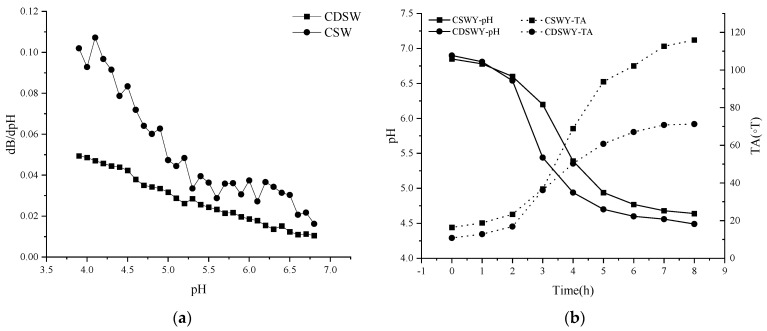
(**a**) Buffering capacity of soybean whey and (**b**) pH and TA changes of soybean whey yogurt as a function of fermentation time. CSW: concentrated soybean whey; CDSW: concentrated desalted soybean whey; CSWY: concentrated soybean whey yogurt; CDSWY: concentrated desalted soybean whey yogurt.

The buffering capacity of a dairy product or non-dairy product is an important physicochemical property related to its ability to be acidified or alkalized. Buffering capacity is related to the minor components and the proteins [27]. As shown in Figure 1a, the buffering capacity of CSW was generally higher than that of CDSW throughout the acidification process. The buffering capacities of both were close in the pH range of 5.0 to 6.9. In the range of 3.9 to 5.0, the buffering capacity of the two differed significantly. SW with high electrical conductivity (~8.2 mS/cm) contained a certain amount of anions and cations. Under the action of electrodialysis, most of the salt ions were removed; thus, the pH change of the CDSW was relatively significant during the acidification process because there was more OH^−^ in the CSW than in the CDSW.

#### 3.2.2. Changes in pH and TA during Fermentation

The pH and TA changes of soybean whey during fermentation are shown in Figure 1b. LAB used carbon sources such as glucose and sucrose to produce organic acids, leading to a gradual decrease in pH. The time to reach the end point of fermentation (~pH 4.5) was 8 h for CDSWY. On the contrary, CSWY reached a final pH of 4.64 after 8 h of fermentation and reached close to the fermentation endpoint after 12 h (pH 4.58). Parvarei et al. [28] studied the chemical changes during fermentation. They found that there are five distinct phases in the fermentation process, in chronological order: lag phase, pre−log phase, log phase, late phase and stable phase. According to this theory, the bacteria were in the lag phase in the first 2 h of fermentation. The pH decreased rapidly and the titratable acidity also increased rapidly (2~5 h) when they were in the pre-logarithmic and logarithmic phases. After this period, due to the gradual depletion of the carbon and nitrogen sources, the fermentation entered a stabilization period and the pH decreased slowly (5~8 h).

CDSW had a lower buffering capacity than CSW, thus the pH dropped faster during fermentation. From the results of LAB count in yogurt, the numbers of LAB in CSWY and CDSWY were 1.9 × 10^7^ CFU/g and 3.7 × 10^8^ CFU/g (Supplementary Materials, Table S1), respectively. Under the action of high concentration of salt stress, the LAB cell membrane structure is damaged, which leads to various intracellular metabolic disorders and even cell death [29,30]. Obviously, too much salt in CSW limited the growth of LAB and lowered the fermentation rate. It should be noted that, during the same fermentation time of 8 h, the titratable acidity of CSWY was obviously higher than that of CDSWY (115.90 vs. 67.07 ◦T), which might be due to the high free amino acid content of CSW, especially the key amino acids arginine, lysine and glutamic acid (Supplementary Materials, Table S2), which facilitate the production of acid [31].

### 3.3. Protein Compositions of Soybean Whey Proteins after Desalination, Concentration and and Fermentation

**Figure 2:**
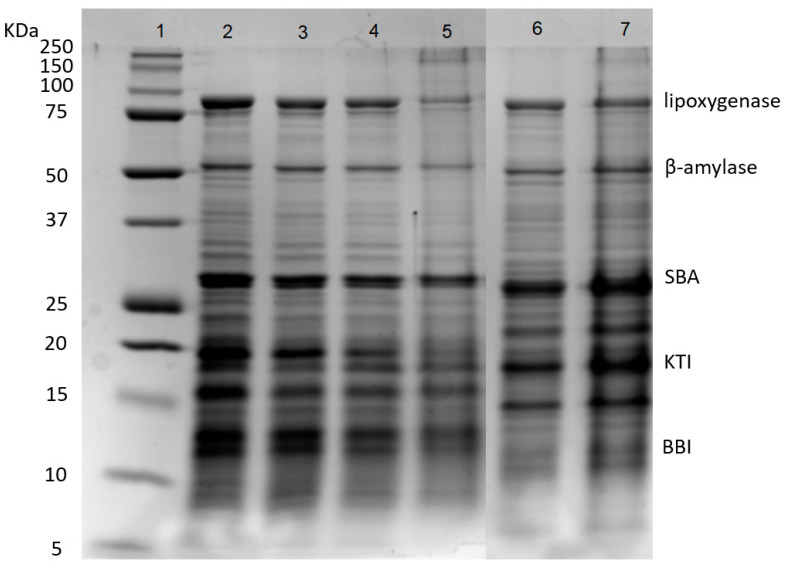
SDS-PAGE of proteins during desalination, concentration and fermentation. Lanes 1–7: marker, SW, DSW, CDSW, CDSWY, CSW, CSWY, respectively. SBA: Soybean agglutinin; KTI: Kunitz trypsin inhibitor; BBI: Bowman–Birk trypsin inhibitor.

Soybean whey proteins were mainly composed of 2S and 7S proteins. As shown in Figure 2, SWPs mainly contained five proteins, including lipoxygenase (7S, 102 kDa), β-amylase (7S, 61.7 kDa), SBA (7S, 120 kDa), KTI (2S, 20 kDa) and BBI (2S, 7.9 kDa). The SWPs had weak gelation properties due to the lack of 11S protein because the gels formed by 11S globulins are harder than those formed by 7S globulins [32]. The results showed that the desalination and concentration treatments had few impacts on the protein composition of SWPs. After fermentation, the partial degradation of lipoxygenase and β-amylase was observed (lanes 5 and 7), due to the hydrolysis of proteins by lactic acid bacteria. In addition, protein aggregates appeared at the top of lane 5, because heat sterilization resulted in the denaturation and aggregation of protein molecules [33,34].

### 3.4. Textural Property of Soybean Whey Yogurt

The textural property of yogurt was mainly characterized by firmness, consistency, cohesiveness and work of cohesion. The textural parameters of the two yogurts are shown in Table 2. There were significant differences (*p* < 0.05) in firmness, consistency, cohesiveness and work of cohesion between the two yogurts. Apparently, the firmness and consistency of CDSWY were much greater than that of CSWY. The gel formation of the yogurt was a kind of acid-induced gel through the accumulation of organic acids during the growth of LAB. With the pH decreasing, the net charge of the protein is neutralized, leading to the formation of a protein gel. The properties of protein gels are influenced by many factors, including protein conformations, heating temperature and salt concentration [35]. The high ionic concentration of the original soybean whey not only affects the rate of fermentation but also the strength of the protein gel. The high ionic concentration could screen the electrostatic interactions and increase the denaturation temperature of the protein, resulting in a lower gel hardness. Nagano et al. [36] reported that the gelation time of 7S globulins increased and the gelation rate became slower with increasing NaCl concentration, which may have been caused by the denaturation temperature of 7Ss protein moving to higher values with increasing ionic strength. Consequently, the removal of salt in soybean whey significantly improved the gel strength of yogurt.

### 3.5. Microstructure of Soybean Whey Yogurt

**Figure 3:**
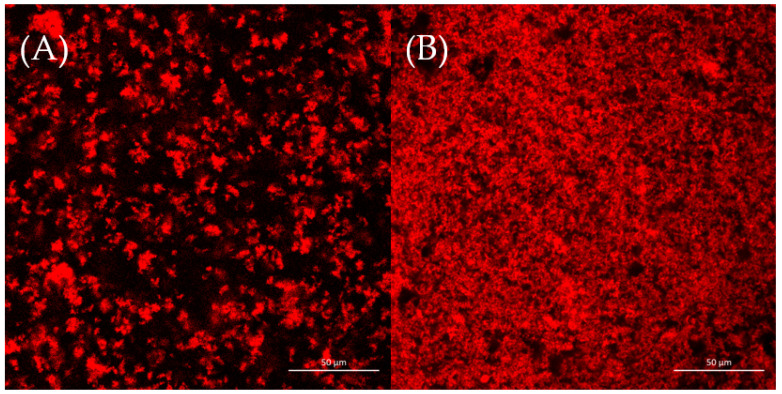
The CLSM images (×40) of (**A**) CSWY and (**B**) CDSWY. Scale bars: 50 μm.

The microstructures of two yogurt samples were observed by CLSM, as shown in Figure 3. It was reported that aggregation and phase separation played important roles in the formation of protein gel network microstructures [37]. Figure 3 shows that the CSWY had a rough and loose network; inversely, CDSWY had a homogeneous, stronger dense gel microstructure. At a high salt concentration, the repulsion between protein molecules was enhanced due to stronger hydration, which resulted in a less dense gel structure in CSWY [38]. The homogeneous dense gel microstructure facilitated the gel strength of CDSWY, while the rough and loose structure of CSWY explained why its textural property was weak.

### 3.6. Gelation Kinetics and Viscoelastic Property of Soybean Whey Yogurt

**Figure 4:**
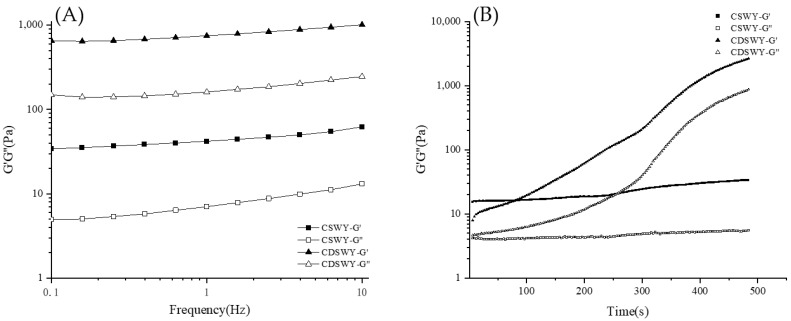
Changes in G′ and G″ of CSWY and CDSWY during (**A**) frequency sweep and (**B**) time sweep.

Frequency sweep is used to characterize the viscoelastic properties of yogurt gels. As shown in Figure 4A, both yogurts had larger G′ values than G″ values, indicating that the yogurts exhibit mainly solid-like properties. Notably, the G′ and G″ of CDSWY were consistently higher than those of CSWY, indicating that CDSWY had a higher gel strength with stronger protein interactions. At a high salt concentration, the salt ions had an electrostatic shielding effect on the protein, reducing the solubility of the protein and thus reducing the hydrogen bonding and electrostatic interaction between the protein molecules and carrageenan. As a result, the solid-like properties of the composite gel were weakened. These results were similar to the findings reported by Zhou et al. [39,40]. As shown in Figure 4B, during the acidification process, the G′ and G″ of CSWY increased very slowly with the increase in incubation time. However, the G′ and G″ of CDSWY increased rapidly, and the final G’ was significantly higher than that of CSWY. A larger G’ represented a stronger gel network formed, which represented a stronger binding of molecules in the CDSWY during the gel formation [41]. Thus, the removal of salt in soybean whey promoted the gel formation, while a high salt concentration inhibited the gel formation during fermentation.

### 3.7. Activities of Urease and Trypsin Inhibitors

Two trypsin inhibitors (KTI and BBI) are the main anti-nutritional factors of soybean whey. Trypsin inhibitors are able to inhibit the activity of trypsin, which affects the absorption of protein by the body; meanwhile, more trypsin is secreted to replenish the intestine, resulting in the waste of endogenous protein in the body [42]. The inactivation of urease is similar to the inactivation of trypsin inhibitors and, therefore, urease activity is mostly used to indicate the removal of trypsin inhibitors [43]. As shown in Table 3, the urease activity of SW was negative after being concentrated regardless of desalination or lack thereof. Urease activity of both yogurts (CSWY and CDSWY) was also not detected. However, for the same CSW or CDSW, only about 30% of trypsin inhibitory activity was inactivated, which was not consistent with the result of urease activity. It has been reported that trypsin inhibitors have high thermal stability, and their inactivation requires high temperatures [44]. During the concentration process, the temperature of 70 °C was not high enough to completely inactivate the trypsin inhibitors. For PCSW and PCDSW, the pasteurization resulted in a further ~40% reduction in TIA. The fermentation resulted in a ~20% further reduction in TIA, possibly due to the increased bacterial protease activity during fermentation. A similar reduction in TIA was also reported for pasteurized and fermented yellow field peas, where they thought it was mainly due to a combination effect of thermal inactivation and the hydrolysis of proteases [45]. Eventually, over 90% of trypsin inhibitory activity was inactivated for CSWY and CDSWY with respect to the initial samples (SW and DSW). The above results show that the heating and fermentation treatments can significantly lower the level of urease activity and trypsin inhibitory activity of CSWY and CDSWY.

### 3.8. Volatile Flavor Compounds of Soybean Whey after Desalination, Concentration and Fermentation

**Figure 5:**
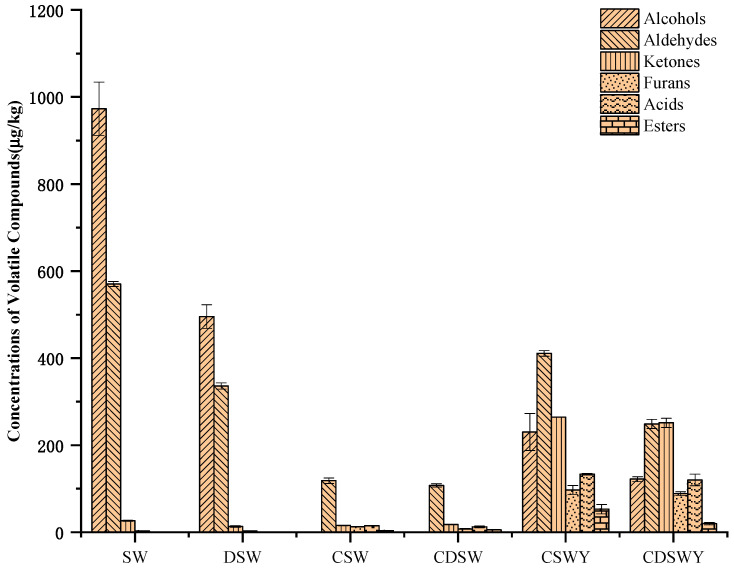
Content of volatile flavor compounds in SW, DSW, CSW, CDSW, CSWY and CDSWY.

The contents of volatile compounds in different treated soybean whey and yogurt are shown in Table 4. Generally, the volatile compounds were identified by HS-SPME-GC/MS, which could be classified as alcohols, aldehydes, ketones, furans, acids and esters. Aldehydes and alcohols were the main components in SW (Figure 5). The total content of volatile compounds in DSW was significantly reduced after desalination. It was estimated that over 46.06% of volatile compounds were lost, including 30.32% alcohols, 14.90% aldehydes and 0.84% ketones. The loss in volatile compounds has been previously observed for mussel cooking juices during desalination by electrodialysis [46]. According to their work, the diffusion or electrotransport of volatile compounds through the ion-exchange membranes or their adsorption onto the membranes led to the loss of volatile compounds. In addition, in our case, the loss of amino acids (Supplementary Materials, Table S2) might induce the co-precipitation of volatile compounds; meanwhile, the ionization of volatile compounds at the very low salt concentration at the end of electrodialysis also cannot be excluded.

For CSW, there was also a significant decrease (89.62%) in the total content of volatile compounds compared with SW, which mainly included alcohols and aldehydes. Similar results have been observed for apple and grape juices [47,48] and sucrose solutions [49]. Compared with desalination by electrodialysis, the vacuum evaporation concentration can easily volatilize flavor substances, especially at high heating temperatures reaching the boiling point of flavor substances. For CDSW, the decrease in volatile compounds was over 91.44% due to the combination effect of desalination and vacuum evaporation concentration.

Different from the loss of flavor components in fruit juice, the volatile compounds lost in soybean whey were mainly beany off-flavors, such as 1-octene-3-ol, hexanal, (E)-2-nonenal and 1-octen-3-one. These compounds make soybean whey have a mushroom flavor, grass flavor and earth flavor. 1-octene-3-ol and 1-octen-3-one were octacarbon compounds. 1-octen-3-one was derived from the oxidative cracking of polyunsaturated fatty acids [50]. 1-octene-3-ol was derived from the enzymatic hydrolysis of alcoholic glycosides in soybean hypocotyls [51]. A reduction in these beany flavors by desalination and vacuum concentration will be beneficial to the improvement of the fermented product flavor. Compared with soybean whey, for the fermented soybean whey yogurts CSWY and CDSWY, the hexanal was reduced by 95.81% and 96.19%, 1-octene-3-ol was reduced by 99.68% and 99.79%, and 1-octen-3-one was reduced by 100% and 100%, respectively. It should be noted that some aldehydes and furans that were not detected or detected at very low levels in the original, desalted and concentrated soybean whey, including (E,E)-2,4-decadienal, (E,E)-2,4-Heptadienal, 2,4-decadienal and 2-pentylfuran, increased significantly after fermentation. These accumulated aldehydes and furans in soybean whey yogurts might be the oxidation products of linolenic acid and linoleic acid of sunflower oil through a residual lipoxygenase (LOX)-induced enzymatic oxidation reaction [52]. In addition, 2,3-butanedione, 2,3-pentanedione, 3-Hydroxy-2-butanone and 2-heptanone were the characteristic creamy or fruity flavors in fermented soybean whey yogurt.

Odor Activity Values (OAVs) were introduced to assess the contribution of key volatile odor compounds to the flavor of a sample. Only flavor compounds that exceed the odor threshold, i.e., OAVs > 1, can contribute to the overall flavor [53]. As shown in Table 5a, the top three key substances in terms of relative contribution rate of the SW and DSW were 1-octen-3-one, 1-octen-3-ol and hexanal, which are typical off-flavors with mushroomy and grassy flavors [8]. On the contrary, in the case of concentrated soybean whey (CSW and CDSW), the 1-octen-3-one and 1-octen-3-ol disappeared; meanwhile, nonanal, β-damascenone and (E)-2-nonenal showed high OAVs and contributed significantly to the flavor (Table 5b). The presence of β-damascenone was first detected in the treated soybean whey. It had not been previously reported in soybean whey but is well recognized as an important aromatic compound with a pleasant fragrance. β-damascenone, a C13-norisoprenoid, is a product naturally formed by the hydrolytic breakdown of complex secondary metabolites derived from carotenoids [54]. It seemed that the degradation of soybean carotenoids occurred due to heat treatment during the vacuum evaporation concentration. The 6-methyl-5-heptene-2-one detected in CSW and CDSW was also considered to be the product of carotenoid degradation [55].

After fermentation, the volatile components in the yogurt were significantly different from the original and treated soybean whey. As shown in Table 5c, the most significant flavor-contributing compound for both CSWY and CDSWY was 2,3-butanedione (relative contributions of 58.38% and 65.47%, respectively). LAB could metabolize citric and pyruvic acid during fermentation to produce 2,3-butanedione and 3-hydroxy-2-butanone [56]. 2,4-decadienal, (E,E)-2,4-decadienal and β-damascenone were the other dominant contributing compounds in both yogurts. The high contribution of 2,4-decadienal was highly related to the oxidative breakdown products of linolenic acid and linoleic acid of sunflower oil. The high contribution of 2,3-butanedione and the presence of β-damascenone improved the flavor characteristics of yogurt.

In our study, the improved volatile flavors of CSWY and CDSWY were reflected in the reduction in off-flavors and the increase in pleasant flavors. Compared with other SW-based yogurts published in the literature, there are both similarities and differences in terms of volatile flavors. For example, Xu et al. [6] prepared soy protein yogurt containing SW and reported that 1-octen-3-ol, hexanal and 3-Hydroxy-2-butanone were the main volatile compounds in their yogurt. However, in our study, the most significant contributing flavor compounds were 2,3-butanedione, 2,4-decadienal, (E,E)-2,4-decadienal and β-damascenone, because desalination and vacuum evaporation concentration significantly reduced the content of 1-octen-3-ol and hexanal. The formation of 2,3-Butanedione [57] and β-damascenone brought creamy and rose aromas to CDSWY, but they were not detected in their work, possibly due to the different processing method and probiotic microorganism addition. Xu et al. [58] fermented mixtures containing SPI and SW using Danisco mixed *lactobacillus* and *B. lactis* HCS04-001, respectively. According to their work, the relative content of (E, E)-2,4-decadienal and 1-octen-3-ol was over 33% and 32%, respectively, which was obviously higher than that in our CDSWY (about 1.17% and 0.15% calculated from Table 4). Tu et al. [5] prepared a novel functional beverage produced from soy whey using water kefir grains, and their results showed that 2-pentyl furan and hexanal were completely metabolized; meanwhile, some new aromatic volatile compounds were produced. In contrast, 2-pentyl furan and hexanal can still be detected in our work, although their OAVs were low. This might be attributed to the different metabolic capacity of water kefir grains in a dilute beverage system, but the fermentation and flavor improvement effect of water kefir grains in a high-protein yogurt system still requires further investigation. 

**Table 5:** (a). The OAVs of key odor-active volatiles and their contribution rates in SW and DSW. (b). The OAVs of key odor-active volatiles and their contribution rates in CSW and CDSW. (c). The OAVs of key odor-active volatiles and their contribution rates in CSWY and CDSWY.

(a)								
Compounds	Odor Description	Threshold (μg/kg in Water)	SW	OAVs	Relative Contribution Rate (%)	DSW	OAVs	Relative Contribution Rate (%)
			(μg/kg			(μg/kg		
			Sample)			Sample)		
1-Octen-3-one	Mushroom, earthy, fruity	0.007	5.92 ± 0.43	845.40 ± 62.02	60.41 ± 3.29	6.87 ± 0.00	981.89 ± 0.46	77.79 ± 0.74
1-Octen-3-ol	Mushroom	1.5	570.29 ± 66.16	380.19 ± 44.10	27.22 ± 3.67	301.80 ± 20.17	201.20 ± 13.45	15.94 ± 0.92
Hexanal	Green, grass	4.5	474.82 ± 5.77	105.52 ± 1.28	7.55 ± 0.23	284.87 ± 14.15	63.31 ± 3.15	5.02 ± 0.29
(E,E)-2,4-Nonadienal	Fat, fried	0.1	2.99 ± 1.17	29.92 ± 11.74	2.13 ± 0.80	-	-	-
(E)-2-Nonenal	Grease, tallow, grass	0.19	4.28 ± 0.68	22.52 ± 3.58	1.61 ± 0.29	1.73 ± 0.25	9.08 ± 1.30	0.72 ± 0.10
Nonanal	Wax, citrus, fat, flowers	1	5.25 ± 0.54	5.25 ± 0.54	0.38 ± 0.03	2.96 ± 0.08	2.96 ± 0.08	0.23 ± 0.01
Heptanal	Grease, grass	2.8	13.61 ± 1.94	4.86 ± 0.69	0.35 ± 0.04	7.60 ± 0.59	2.71 ± 0.21	0.21 ± 0.01
(E)-2-Octenal	Cucumber, vegetable	3	5.99 ± 1.07	2.00 ± 0.36	0.14 ± 0.02	3.39 ± 0.54	1.13 ± 0.18	0.09 ± 0.01
Octanal	Lemon and fruit	0.8	1.33 ± 0.07	1.66 ± 0.09	0.12 ± 0.00	-	-	-
Pentanal	Fermentation, yogurt	12	16.72 ± 0.15	1.39 ± 0.01	0.10 ± 0.00	9.04 ± 1.62	<1	-
**(b)**								
**Compounds**	**Odor Description**	**Threshold (μg/kg in Water)**	**CSW** **(μg/kg** **Sample)**	**OAVs**	**Relative Contribution Rate (%)**	**CDSW** **(μg/kg** **Sample)**	**OAVs**	**Relative Contribution Rate (%)**
(E)-2-Nonenal	Grease, tallow, grass	0.19	5.15 ± 0.23	27.12 ± 0.59	27.31 ± 1.76	2.83 ± 0.24	14.89 ± 1.27	20.23 ± 0.71
β-damascenone	Apples, roses, honey	0.056	1.19 ± 0.11	21.32 ± 1.01	21.44 ± 1.59	0.44 ± 0.09	7.81 ± 1.62	11.62 ± 1.79
Nonanal	Wax, citrus, fat, flowers	1	18.95 ± 0.99	18.95 ± 0.49	19.06 ± 0.60	25.09 ± 0.64	25.09 ± 0.64	37.56 ± 2.98
(E,E)-2,4-Nonadienal	Fat, fried	0.1	0.77 ± 0.03	7.66 ± 0.15	7.71 ± 0.47	0.64 ± 0.09	6.41 ± 0.90	9.55 ± 0.82
Hexanal	Green, grass	4.5	27.28 ± 0.44	6.06 ± 0.05	6.10 ± 0.23	10.94 ± 0.80	2.43 ± 0.18	3.64 ± 0.46
(E,E)-2,4-Decadienal	Fried, wax, fat	0.07	0.34 ± 0.04	4.80 ± 0.32	4.83 ± 0.54	-	-	-
2,4-Decadienal	Fat	0.3	1.02 ± 0.02	3.39 ± 0.04	3.41 ± 0.15	0.81 ± 0.01	2.69 ± 0.04	4.02 ± 0.12
(E)-2-Octenal	Cucumber, vegetable	3	7.88 ± 0.34	2.63 ± 0.06	2.64 ± 0.17	4.91 ± 0.28	1.64 ± 0.09	2.45 ± 0.27
Octanal	Lemon and fruit	0.8	1.91 ± 0.11	2.38 ± 0.07	2.40 ± 0.19	2.10 ± 0.24	2.62 ± 0.30	3.91 ± 0.24
(E)-2-Heptenal	Soap, fat, almond	13	25.62 ± 5.32	1.97 ± 0.20	1.98 ± 0.37	20.42 ± 4.83	1.57 ± 0.37	2.33 ± 0.43
2-Pentylfuran	Grass, beany, butter	6	10.29 ± 0.08	1.72 ± 0.01	1.73 ± 0.05	5.50 ± 0.56	<1	-
Decanal	Earthy, mushroom	3	4.15 ± 0.19	1.38 ± 0.03	1.39 ± 0.09	5.38 ± 0.06	1.79 ± 0.02	2.68 ± 0.11
**(c)**								
**Compounds**	**Odor Description**	**^a^** **Threshold (μg/kg in Water)**	**CSWY** **(μg/kg** **Samples)**	**OAVs**	**Relative Contribution Rate (%)**	**CDSWY** **(μg/kg** **Samples)**	**OAVs**	**Relative Contribution Rate (%)**
2,3-Butanedione	Butter	0.059	70.05 ± 0.86	1187.34 ± 14.62	58.38 ± 0.68	78.92 ± 6.06	1337.64 ± 102.63	65.47 ± 1.11
2,4-Decadienal	Fat	0.3	122.11 ± 19.50	407.04 ± 64.99	19.83 ± 2.92	112.93 ± 2.87	376.43 ± 9.58	18.45 ± 0.63
(E,E)-2,4-Decadienal	Fried, wax, fat	0.07	16.00 ± 0.49	228.55 ± 6.94	11.15 ± 0.48	10.47 ± 0.26	149.59 ± 3.67	7.34 ± 0.62
β-damascenone	Apples, roses, honey	0.056	3.40 ± 0.10	60.78 ± 1.84	2.96 ± 0.05	2.22 ± 0.16	39.73 ± 2.86	1.94 ± 0.02
(E,E)-2,4-Nonadienal	Fat, fried	0.1	4.04 ± 1.03	40.36 ± 10.32	1.97 ± 0.53	3.07 ± 0.21	30.73 ± 2.15	1.50 ± 0.02
(E)-2-Decenaldehyde	Chicken oil, oranges	0.3	7.14 ± 0.76	23.81 ± 2.54	1.16 ± 0.11	8.07 ± 0.26	26.89 ± 0.87	1.32 ± 0.04
(E)-2-Nonenal	Grease, tallow, grass	0.19	6.85 ± 1.12	36.04 ± 5.89	1.75 ± 0.22	5.56 ± 1.48	29.25 ± 7.78	1.42 ± 0.30
2-Pentylfuran	Grass, beany, butter	6	91.05 ± 9.78	15.18 ± 1.63	0.74 ± 0.09	80.77 ± 2.72	13.46 ± 0.45	0.66 ± 0.02
(E)-2-Heptenal	Soap, fat, almond	13	175.26 ± 14.92	13.48 ± 1.15	0.66 ± 0.06	34.93 ± 0.48	2.69 ± 0.04	0.13 ± 0.01
Nonanal	Wax, citrus, fat, flowers	1	12.85 ± 7.28	12.85 ± 7.28	0.62 ± 0.35	15.91 ± 0.71	15.91 ± 0.71	0.78 ± 0.08
(E)-2-Octenal	Cucumber, vegetable	3	14.81 ± 1.37	4.94 ± 0.46	0.24 ± 0.03	14.81 ± 1.37	4.94 ± 0.46	0.20 ± 0.01
Hexanal	Green, grass	4.5	19.90 ± 0.26	4.42 ± 0.06	0.22 ± 0.01	18.08 ± 2.16	4.02 ± 0.48	0.20 ± 0.01
Octanal	Lemon and fruit	0.8	3.34 ± 0.71	4.18 ± 0.89	0.20 ± 0.04	2.78 ± 0.00	3.47 ± 0.00	0.17 ± 0.01
2,3-Pentanedione	Cream, butter	20	76.48 ± 6.34	3.82 ± 0.32	0.19 ± 0.02	45.28 ± 1.56	2.26 ± 0.08	0.11 ± 0.00
Ethyl hexanoate	Fruit	5	16.92 ± 5.19	3.38 ± 1.04	0.17 ± 0.05	2.40 ± 0.80	<1	-
3-Hydroxy-2-butanone	Cream, fat	14	26.04 ± 1.60	1.86 ± 0.11	0.09 ± 0.01	55.99 ± 0.73	4.00 ± 0.05	0.20 ± 0.01
1-Octen-3-ol	Mushroom	1.5	1.83 ± 0.12	1.22 ± 0.08	0.06 ± 0.00	1.17 ± 0.02	<1	-
Heptanal	Grease, grass	2.8	-	-	-	3.02 ± 0.16	1.08 ± 0.06	0.05 ± 0.01

“-” means not detected in samples. **^a^** Threshold values data from published references [59,60]. The mean ± standard error (*n* = 3) was shown.

### 3.9. Sensory Evaluation

**Figure 6:**
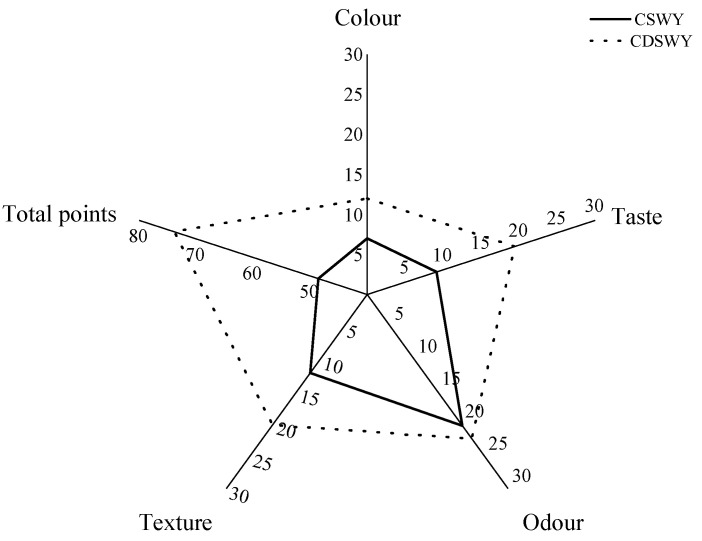
Sensory evaluation of soy whey yogurt. CSWY is concentrated soybean whey yogurt and CDSWY is concentrated desalted soybean whey yogurt.

The color, taste and texture of the soybean whey yogurt differed considerably, as shown in Figure 6. In terms of color, both yogurts were creamy yellow in color, but the CSWY was darker. In terms of taste, CSWY exhibit a distinct salty taste, while the CDSWY was less salty and moderately sweet and sour when tasted. As for the texture, the CDSWY formed a firmer gel and tasted less grainy compared with CSWY. There was no significant difference in odor between both types of yogurts, which was consistent with the volatile flavor compound results. In conclusion, the CDSWY scored the highest, which is consistent with the findings of Mudgil et al. [61] and Xu et al. [6], who showed that higher scores on sensory attributes were associated with enhanced textural properties. The results showed that the removal of salt and off-flavors by desalination, concentration and fermentation by Danisco mixed LAB significantly improved the sensory quality of soybean whey yogurt. This is consistent with the study by Xu et al. [6], who fermented yogurt separately through a variety of probiotics and scored high in sensory scores when fermented with a Danisco fermentation of mixed probiotics.

## 4. Conclusions

In conclusion, desalination and concentration treatments of soybean whey showed a significant impact on the quality of final yogurt samples. The removal of salt ions through electrodialysis desalination was an effective strategy to improve the fermentation rate and LAB count of soybean whey. Vacuum concentration not only increased the protein concentration but also significantly reduced the urease activity and trypsin inhibitory activity of soybean whey. Compared with CSWY, CDSWY exhibited higher hardness, consistency and G’/G’’ values, forming a homogeneous dense gel microstructure. Over 90% of trypsin inhibitory activity was inactivated for CDSWY and CSWY through the combination effect of heating and protein degradation due to concentration and LAB fermentation degradation. Over 90% of off-flavors in soybean whey, including hexanal, 1-octen-3-ol and 1-octen-3-one, were removed after desalination, concentration and fermentation treatments. Meanwhile, the newly generated key pleasant flavors 2,3-butanedione and β-damascenone through carotenoid degradation obviously improved the flavor characteristics and sensory quality of CDSWY, while the salty taste lowered the sensory quality of CSWY. This study provides useful guidance on the utilization of soybean whey in fermented yogurt.

## Figures and Tables

**Table 1:** Basic composition of different desalted or concentrated soybean whey.

Samples	SW	DSW	CSW	CDSW
Solid (%)	3.27 ± 0.02 ^c^	2.22 ± 0.01 ^d^	18.98 ± 0.01 ^a^	17.09 ± 0.02 ^b^
Protein (%)	0.47 ± 0.02 ^b^	0.36 ± 0.02 ^c^	2.50 ± 0.00 ^a^	2.50 ± 0.00 ^a^
Carbohydrate (%)	1.65 ± 0.03 ^c^	1.41 ± 0.03 ^d^	8.94 ± 0.01 ^b^	9.75 ± 0.12 ^a^
Ash (%)	0.40 ± 0.02 ^c^	0.09 ± 0.00 ^d^	2.37 ± 0.25 ^a^	0.76 ± 0.06 ^b^
Sugars (mg/mL sample)				
Fructose	0.91 ± 0.00 ^c^	0.82 ± 0.02 ^c^	4.21 ± 0.06 ^b^	4.68 ± 0.15 ^a^
Sucrose	11.31 ± 0.19 ^c^	8.53 ± 0.05 ^c^	62.51 ± 4.47 ^b^	69.82 ± 0.99 ^a^
Raffinose	1.30 ± 0.04 ^c^	0.91 ± 0.00 ^d^	5.33 ± 0.32 ^b^	6.54 ± 0.29 ^a^
Stachyose	6.79 ± 0.02 ^b^	5.92 ± 0.03 ^c^	10.16 ± 0.46 ^a^	10.60 ± 0.19 ^a^

Where SW is soybean whey, DSW is desalted soybean whey, CSW is concentrated soybean whey and CDSW is concentrated desalted soybean whey. Values in the same row with different letters differ significantly (*p* < 0.05).

**Table 2:** Texture parameters of CSWY and CDSWY.

Yogurt Samples	Firmness/ (g)	Consistency/ (g·s)	Cohesiveness/ (g)	Work of Cohesion/ (g·s)
CDSWY	432.42 ± 37.70 ^a^	2289.40 ± 139.81 ^a^	−14.51 ± 1.22 ^b^	−24.77 ± 1.16 ^b^
CSWY	33.49 ± 1.96 ^b^	187.2 ± 15.78 ^b^	−7.12 ± 0.17 ^a^	−16.78 ± 0.39 ^a^

Values in a column with different letters differ significantly (*p* < 0.05). CSWY is concentrated soybean whey yogurt; CDSWY is concentrated desalted soybean whey yogurt.

**Table 3:** Urease and trypsin inhibitor activities of different samples.

Samples	Urease Activity	Trypsin Inhibitory Activity (U/mg Protein)
SW	+	615.42 ± 54.34
DSW	+	699.67 ± 6.73
CSW	−	469.70 ± 11.08
CDSW	−	538.54 ± 1.74
PCSW	−	178.29 ± 1.97
PCDSW	−	201.95 ± 21.46
CSWY	−	56.29 ± 0.28
CDSWY	−	66.50 ± 2.74

Where “+” means positive, “−” means negative, SW is soybean whey, DSW is desalted soybean whey, CSW is concentrated soybean whey, CDSW is concentrated desalted soybean whey; PCSW and PCDSW are CSW and CDSW pasteurized at 95 °C for 10 min; CSWY is concentrated soybean whey yogurt and CDSWY is concentrated desalted soybean whey yogurt.

**Table 4:** Concentrations of volatile compounds (μg/kg) in original soybean whey, desalted soybean whey, concentrated whey and fermented soybean whey yogurts.

Volatile Compounds	SW	DSW	CSW	CDSW	CSWY	CDSWY
Alcohols						
1-Hexanol	233.51 ± 0.22	104.77 ± 1.94	-	-	121.60 ± 39.25	45.48 ± 1.07
1-Octanol	2.13 ± 0.59	-			28.36 ± 3.47	8.12 ± 0.99
1-Pentanol	48.13 ± 4.01	26.90 ± 0.02	-	-	-	-
2-Hexanol	45.00 ± 0.07	24.83 ± 0.93	-	-	-	-
2-Octanol	2.39 ± 0.68	-	-	-	-	-
3-Hexanol	43.99 ± 0.17	23.00 ± 3.25	-	-	-	-
Benzyl alcohol	2.26 ± 0.12	1.37 ± 0.09	-	-	-	-
1-Dodecanol	10.26 ± 0.92	4.66 ± 0.48	-	-	-	-
2-Ethyl-1-hexanol	1.96 ± 0.16	2.42 ± 0.17	-	-	-	-
1-Octen-3-ol	570.29 ± 66.16	301.80 ± 20.17	-	-	1.83 ± 0.12	1.17 ± 0.02
(E)-2-Hepten-1-ol	2.74 ± 0.17	1.70 ± 0.01	-	-	-	-
(E)-2-Penten-1-ol	6.58 ± 0.12	2.91 ± 0.15	-	-	-	-
(E)-3-Hexen-1-ol	1.49 ± 0.61	1.22 ± 0.02	-	-	-	-
1- Nonanol	-	-	-	-	47.80 ± 1.59	61.02 ± 3.97
Phenethyl alcohol	-	-	-	-	11.30 ± 0.08	6.21 ± 0.43
Aldehydes						
Hexanal	474.82 ± 5.77	284.87 ± 14.15	27.28 ± 0.44	10.94 ± 0.80	19.90 ± 0.26	18.08 ± 2.16
Nonanal	5.25 ± 0.54	2.96 ± 0.08	18.95 ± 0.99	25.09 ± 0.64	12.85 ± 7.28	15.91 ± 0.71
Octanal	1.33 ± 0.07	-	1.91 ± 0.11	2.10 ± 0.24	3.34 ± 0.71	2.78 ± 0.00
Pentanal	16.72 ± 0.15	9.04 ± 1.62	-	-	-	-
Heptanal	13.61 ± 1.94	7.60 ± 0.59	2.19 ± 0.08	1.47 ± 0.17	-	3.02 ± 0.16
Decanal	-	1.40 ± 0.06	4.15 ± 0.19	5.38 ± 0.06	-	-
Benzaldehyde	8.41 ± 1.03	4.17 ± 0.77	7.79 ± 0.15	6.86 ± 0.13	18.32 ± 0.26	17.03 ± 0.04
(E)-2-Heptenal	11.60 ± 0.57	6.22 ± 0.28	25.62 ± 5.32	20.42 ± 4.83	175.26 ± 14.92	34.93 ± 0.48
(E)-2-Hexenal	19.10 ± 5.56	11.37 ± 1.72	9.62 ± 0.23	5.03 ± 0.42	8.22 ± 0.54	7.46 ± 0.03
(E)-2-Nonenal	4.28 ± 0.68	1.73 ± 0.25	5.15 ± 0.23	2.83 ± 0.24	6.85 ± 1.12	5.56 ± 1.48
(E)-2-Octenal	5.99 ± 1.07	3.39 ± 0.54	7.88 ± 0.34	4.91 ± 0.28	14.81 ± 1.37	14.81 ± 1.37
(E)-2-Pentenal	6.44 ± 1.27	3.24 ± 1.44	2.76 ± 1.17	2.06 ± 0.09	-	-
(E,E)-2,4-Nonadienal	2.99 ± 1.17	-	0.77 ± 0.03	0.64 ± 0.09	4.04 ± 1.03	3.07 ± 0.21
Phenylacetaldehyde	-	-	1.52 ± 0.10	1.47 ± 0.19	1.63 ± 0.11	2.16 ± 0.32
(E,E)-2,4-Decadienal	-	-	0.34 ± 0.04	-	16.00 ± 0.49	10.47 ± 0.26
(E,E)-2,4-Heptadienal	-	-	1.34 ± 0.17	1.98 ± 0.15	3.38 ± 0.19	4.84 ± 0.11
(E)-2-Decenaldehyde	-	-	-	-	7.14 ± 0.76	8.07 ± 0.26
2,4-Decadienal	-	-	1.02 ± 0.02	0.81 ± 0.01	122.11 ± 19.50	112.93 ± 2.87
Ketones						
3-Octanone	4.91 ± 0.35	-	-	-	1.72 ± 0.93	2.29 ± 0.10
Acetophenone	0.61 ± 0.07	1.14 ± 0.07	1.66 ± 0.58	2.29 ± 0.03	3.35 ± 0.03	5.97 ± 0.79
1-Octen-3-one	5.92 ± 0.43	6.87 ± 0.00	-	-	-	-
(E)-3-Octen-2-one	11.58 ± 1.90	3.62 ± 1.67	5.60 ± 0.14	6.07 ± 0.32	31.95 ± 2.11	33.03 ± 0.14
(E,E)-3,5-Octadien-2-one	3.42 ± 0.15	1.57 ± 0.42	0.71 ± 0.16	2.10 ± 0.17	4.66 ± 0.07	4.23 ± 0.20
2-Nonanone	-	-	0.24 ± 0.02	0.47 ± 0.08	7.54 ± 0.06	8.99 ± 0.61
4- Octanone	-	-	2.00 ± 0.06	2.19 ± 0.39	1.04 ± 0.02	7.23 ± 0.90
2- Undecanone	-	-	0.23 ± 0.01	0.67 ± 0.02	3.24 ± 0.01	2.75± 0.21
β-damascenone	-	-	1.19 ± 0.11	0.44 ± 0.09	3.40 ± 0.10	2.22 ± 0.16
6-Methyl -5-heptene -2- one	-	-	3.81 ± 0.05	3.51 ± 0.02	5.17 ± 0.57	4.40 ± 0.06
2,3-Butanedione	-	-	-	-	70.05 ± 0.86	78.92 ± 6.06
2,3-Pentanedione	-	-	-	-	76.48 ± 6.34	44.87 ± 1.31
3-Hydroxy -2- butanone	-	-	-	-	26.04 ± 1.60	55.99 ± 0.73
2- Heptanone	-	-	-	-	29.68 ± 3.50	18.32 ± 0.08
Furans						
2-Pentylfuran	2.92 ± 0.09	2.57 ± 0.58	10.29 ± 0.08	5.50 ± 0.56	91.05 ± 9.78	80.77 ± 2.72
2- Butylfuran					6.01 ± 0.45	6.96 ± 1.81
2- Ethylfuran			2.16 ± 0.18	1.94 ± 0.46	19.61 ± 1.53	12.97 ± 1.30
Acids						
Acetic acid	-	-	10.03 ± 0.48	6.92 ± 0.76	52.99 ± 1.98	53.46 ± 0.66
Butanoic acid	-	-	0.16 ± 0.02	0.66 ± 0.01	2.67 ± 0.48	1.22 ± 0.34
Hexanoic acid	-	-	1.62 ± 0.37	3.19 ± 0.31	16.16 ± 0.32	30.94 ± 10.43
Decylic acid		-	0.92 ± 0.26	1.26 ± 0.43	2.89 ± 0.91	1.55 ± 0.49
Nonanoic acid	-	-	2.69 ± 0.25	25.50 ± 6.83	34.11 ± 2.58	11.61 ± 3.46
Benzoic acid	-	-	0.88 ± 0.06	0.91 ± 0.10	2.97 ± 0.01	1.48 ± 0.03
Pentanoic acid	-	-	0.61 ± 0.06	0.60 ± 0.01	5.65 ± 0.23	2.84 ± 0.57
Octanoic acid	0.98 ± 0.08	-	-	-	8.35 ± 1.60	8.59 ± 0.52
Heptanoic acid	0.96 ± 0.10	-	-	-	7.47 ± 1.94	8.40 ± 0.54
Esters						
Ethyl hexanoate	-	-	1.52 ± 0.66	0.26 ± 0.07	16.92 ± 5.19	2.40 ± 0.80
Hexyl hexanoate	-	-	0.32 ± 0.04	0.13 ± 0.01	4.32 ± 0.26	1.63 ± 0.27
Ethyl nonanoate	-	-	0.26 ± 0.04	0.22 ± 0.03	2.39 ± 0.90	0.83 ± 0.15
Etheyl octanoat	-	-	1.74 ± 0.52	4.81 ± 0.42	29.33 ± 4.88	14.81 ± 1.37

“-” means not detected in samples.

## Data Availability

The data presented in this study are available on request from the corresponding author.

## Supplementary Materials

The following supporting information can be downloaded at https://www.mdpi.com/article/10.3390/foods13020330/s1, Table S1: Total counts of LAB (CFU/g) for CSWY and CDSWY at different fermentation times; Table S2: Free amino acid content (mg/mL) of concentrated soybean whey (CSW) and concentrated desalted soybean whey (CDSW).
