# Supplementary material for: Fermentation Characteristics, Antinutritional Factor Level and Flavor Compounds of Soybean Whey Yogurt
Source: Foods. 2024 Jan 20;13(2):330. doi: 10.3390/foods13020330 (PMC10814812; doi:10.3390/foods13020330)
Supplement: Supplementary file 1 [file foods-13-00330-s001.zip › foods-2757603-supplementary.pdf]

Supplementary materials

Table S1. Total counts of LAB (CFU/g) for CSWY and CDSWY at different fermentation times

Fermentation time	CSWY	CDSWY
0h (initial point)	$2.2*10^7$	$2.2*10^7$
8h (end point)	$1.9*10^7$	$3.7*10^8$

Table S2. Free amino acids content (mg/mL) of concentrated soybean whey (CSW) and concentrated desalted soybean whey (CDSW).

**Essential amino acid**	**CSW**	**CDSW**
Threonine	$0.07±0.00^a$	$0.05±0.00^b$
Valine	$0.10±0.00^a$	$0.06±0.00^b$
Methionine	$0.02±0.00^a$	$0.02±0.00^a$
Phenylalanine	$0.07±0.00^a$	$0.05±0.00^b$
Isoleucine	$0.05±0.00^a$	$0.04±0.00^b$
Leucine	$0.08±0.00^a$	$0.06±0.00^b$
Lysine	$0.11±0.00^a$	$0.04±0.00^b$
**Non-essential amino acid**		
Aspartic acid	$0.47±0.01^a$	$0.23±0.00^b$
Glutamic acid	$0.55±0.01^a$	$0.34±0.00^b$
Serine	$0.02±0.00^a$	$0.01±0.00^b$
Arginine	$1.66±0.02^a$	$0.30±0.01^b$
Histidine	$0.12±0.00^a$	$0.04±0.00^b$
Glycine	$0.05±0.00^a$	$0.04±0.00^b$
Alanine	$0.16±0.00^a$	$0.12±0.00^b$
Tyrosine	$0.20±0.00^a$	$0.16±0.00^b$
Cysteine	$0.02±0.00^a$	$0.00±0.00^b$
Proline	$0.04±0.01^a$	$0.03±0.01^a$
∑FAAs	$3.78±0.04^a$	$1.59±0.01^b$
